# Supplementary material for: Elevated FAM84B promotes cell proliferation via interacting with NPM1 in esophageal squamous cell carcinoma
Source: Cell Death Discov. 2022 Apr 8;8:182. doi: 10.1038/s41420-022-00984-9 (PMC8993864; doi:10.1038/s41420-022-00984-9)
Supplement: Supplementary file 2 — Table S1-2, Figure S1-8 [file 41420_2022_984_MOESM2_ESM.doc]

SUPPLEMENT TABLES

TABLE S1 Primer used for Realtime-PCR

| **Gene name** | **Forward primer** | **Reverse primer** |
| --- | --- | --- |
| FAM84B | 5′-GACCCAC CTAAGTTACAAGGAAG-3′ | 5′-GT AGAACACGGAGCA TTCCAC-3′ |
| NPM1 | 5′-GAAGAATTGCTTCCGGATGACT-3′ | 5′-GCCAGATATCAACTGTTACAGAAATG-3′ |
| CDKN2A | 5′-GGCTCTACACAAGCTTCCTTTCC-3′ | 5′-TCATGACCTGCCAGAGAGAACA-3′ |
| CDK4 | 5′-ATGGCTACCTCTCGATATGAGC-3′ | 5′-CATTGGGGACTCTCACACTCT-3′ |
| CDK6 | 5′-TCTTCATTCACACCGAGTAGTGC-3′ | 5′-TGAGGTTAGAGCCATCTGGAAA-3′ |
| CCND1 | 5′-CCTCTGTGCCACAGATG-3′ | 5′-GGGTCACACTTGATCACTC-3′ |
| E2F1 | 5′-CTCCTCAGGGCACAGGAA-3′ | 5′-CGTGGACTCTTCGGAGAACTTTC-3′ |
| E2F2 | 5′-CCTTGGAGGCTACTGACAGC-3′ | 5′-CCACAGGTAGTCGTCCTGGT-3′ |
| E2F3 | 5′-GGGCCCATTGAGGTTTACTTATGTC-3′ | 5’-ATCGCTATGTCCTGAGTTGGTTGA-3′ |
| p-Rb | 5′-AAGCCAGTCTTTCCAGAGAATAACC-3′ | 5′-ATCCCAGTCCATCTAGCCTCTTACA-3′ |
| GAPDH | 5′-AAGCCAGTCTTTCCAGAGAATAACC-3′ | 5′-ATCCCAGTCCATCTAGCCTCTTACA-3′ |

TABLE S2 Statistical difference of survival between the four groups divided by FAM84B copy number amplification combined with T stage

| Group | FAM84B_Amp_  + T(Ⅰ+Ⅱ) | | FAM84B_non-Amp_  + T(Ⅰ+Ⅱ) | | FAM84B_Amp_  + T(Ⅲ) | | FAM84B_non-Amp_  + T(Ⅲ) | |
| --- | --- | --- | --- | --- | --- | --- | --- | --- |
|  | χ^2^ | *p^a^* | χ^2^ | *p^a^* | χ^2^ | *p^a^* | χ^2^ | *p^a^* |
| FAM84B_Amp_  + T(Ⅰ+Ⅱ) |  |  | 0.189 | 0.663 | 19.323 | 0.000 | 4.248 | 0.039 |
| FAM84B_non-Amp_  + T(Ⅰ+Ⅱ) | 0.189 | 0.663 |  |  | 46.900 | 7.48E-12 | 16.458 | 0.000 |
| FAM84B_Amp_  + T(Ⅲ) | 19.323 | 0.000 | 46.898 | 7.48E-12 |  |  | 11.154 | 0.001 |
| FAM84B_non-Amp_  + T(Ⅲ) | 4.248 | 0.039 | 16.458 | 0.000 | 11.154 | 0.001 |  |  |

^a^ Log-rank test.

**SUPPLEMENT FIGURES**

**
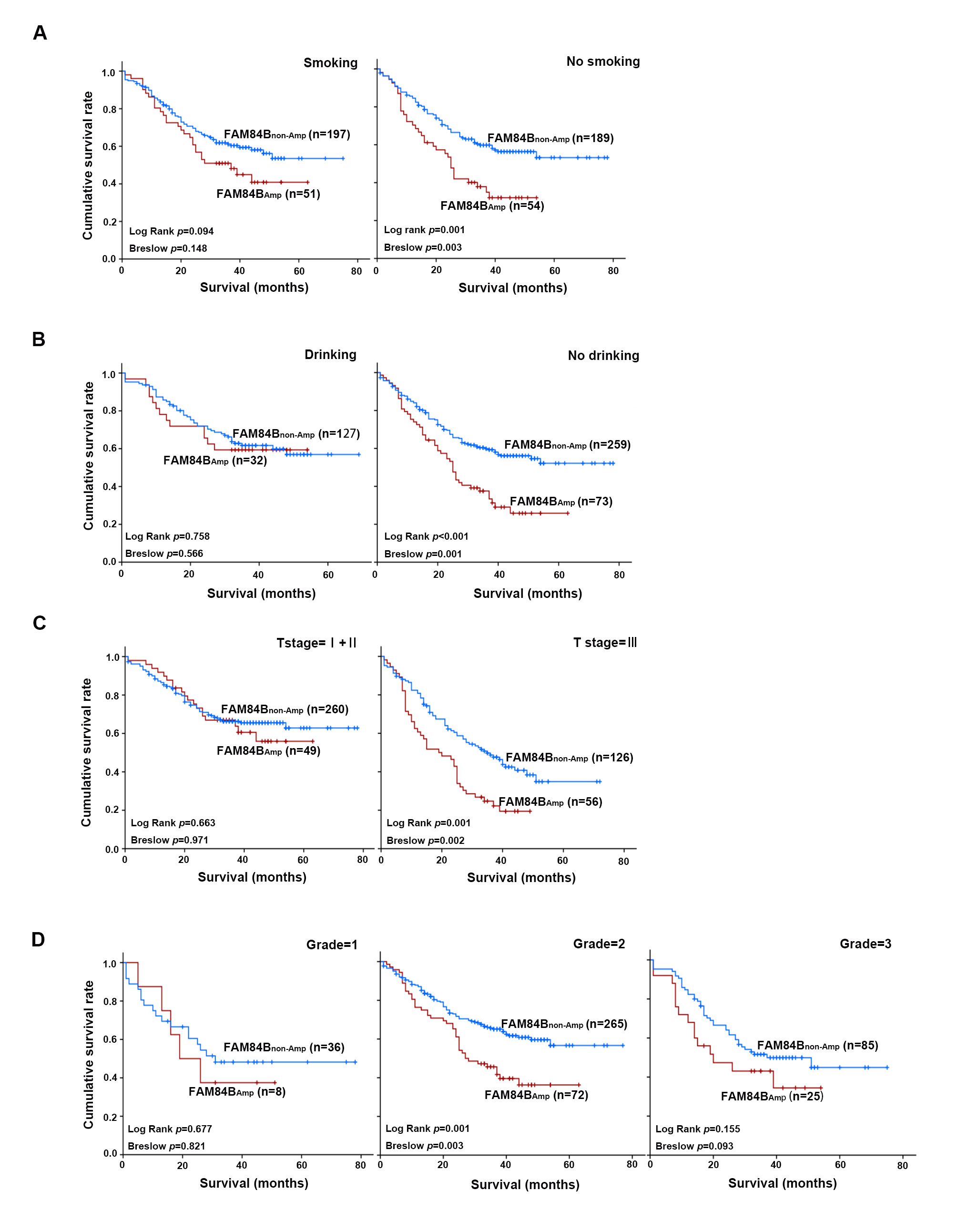
**

**Fig S1 Kaplan-Meier survival plot showed the clinical value of FAM84B copy number amplification in 507 ESCC patients with multiple clinical parameters.**

(A) Kaplan-Meier survival plot showed the cumulative survival rate of 507 ESCC patients with different FAM84B copy number amplification in smoking and no smoking groups. (B) Kaplan-Meier survival plot showed the cumulative survival rate of 507 ESCC patients with different FAM84B copy number amplification in drinking and no drinking groups. (C) Log rank test and Breslow test were used to analyze the survival data of 507 ESCC patients with TNM stage. (D) Log rank test and Breslow test were used to analyze the survival data of 507 ESCC patients with histological grade. *P* < 0.05 was considered statistically significant.

**
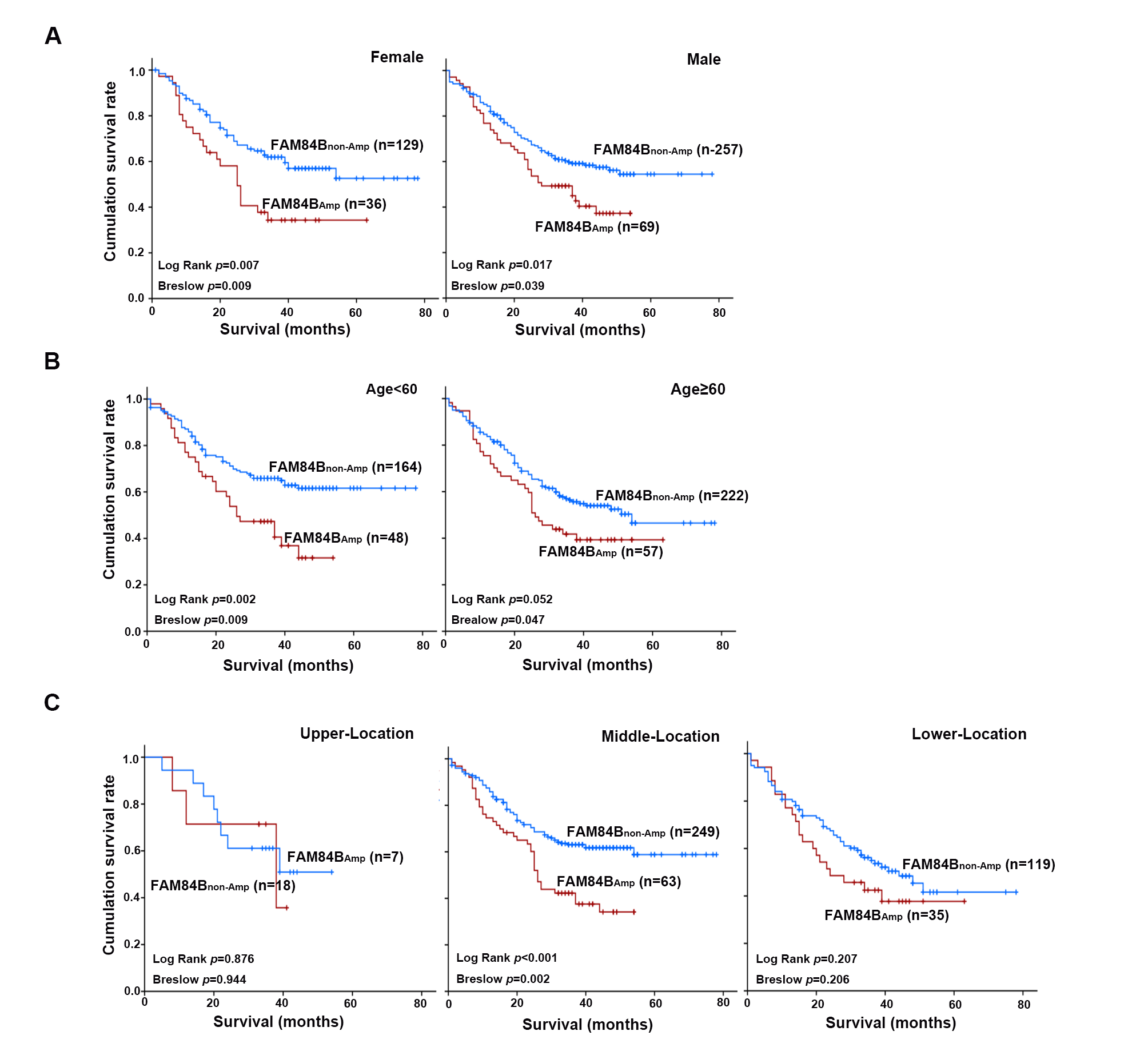
 Fig S2 Kaplan-Meier survival plot showed the clinical value of FAM84B copy number amplification in 507 ESCC patients with different gender, age and location**

(A) Kaplan-Meier survival plot showed the cumulative survival rate of 507 ESCC patients with different FAM84B copy number amplification in male and female groups. (B) Kaplan-Meier survival plot showed the cumulative survival rate of 507 ESCC patients with different FAM84B copy number amplification in Age < 60 and Age ≥ 60 groups. (C) Kaplan-Meier survival plot showed the cumulative survival rate of 507 ESCC patients with different FAM84B copy number amplification in upper, middle and lower location. *P* < 0.05 was considered statistically significant.

**
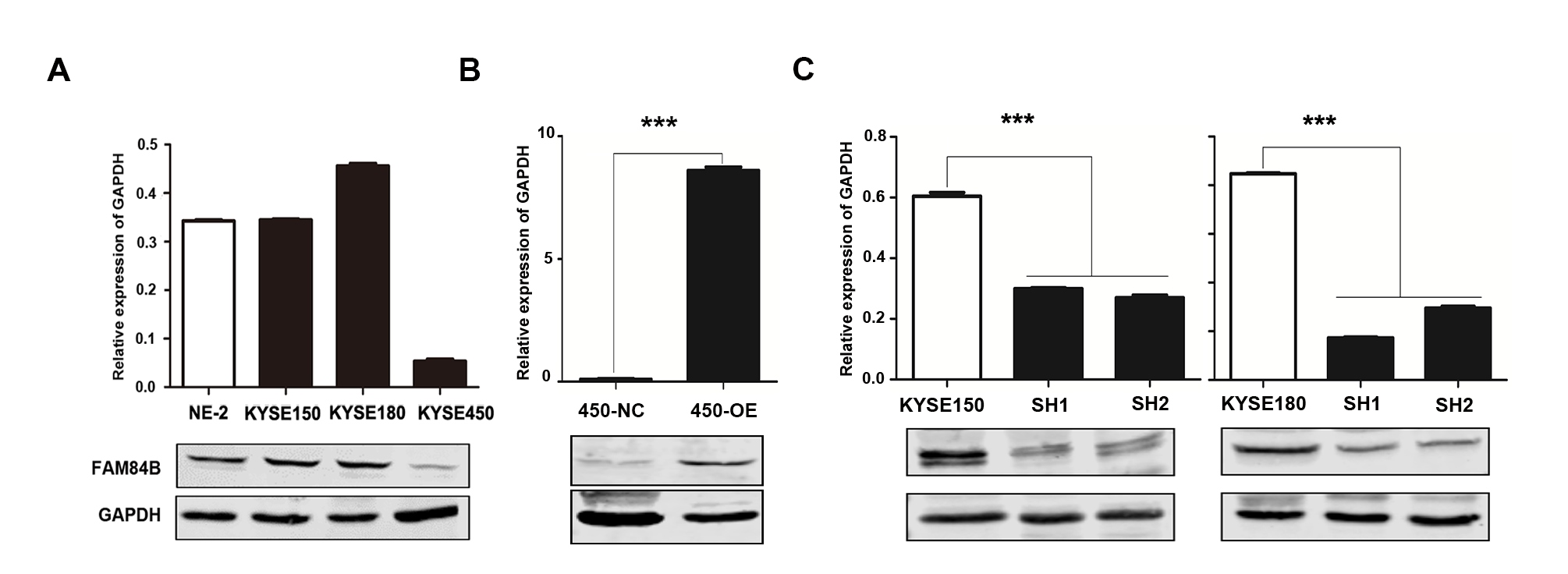
 Fig S3 The expression of FAM84B in ESCC**

(A) The expression levels of FAM84B in ESCC cell lines. (B) Over-expression FAM84B is detected by western blot through transfecting lentivirus in KYSE450 cells. (C) Endogenous FAM84B is knocked down in KYSE150 and KYSE180 cells. Statistical analysis is performed with one-way ANOVA. ****P* < 0.001.


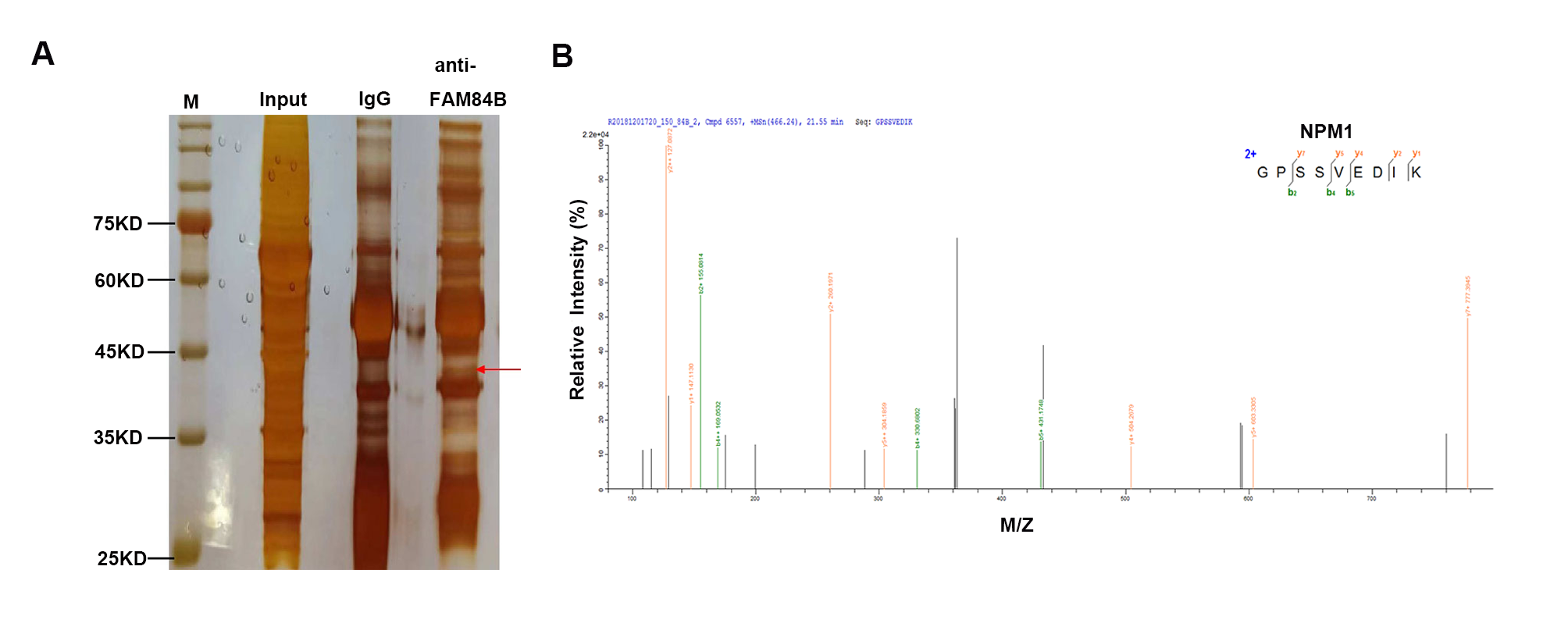


**Fig S4 The interaction and co-location of FAM84B and NPM1**

(A) The cell lysates from 450-OE cells were subjected to Co-IP with the FAM84B antibody, the silver stain photo of control (IgG) and Co-IP (anti-FAM84B) group is shown. (B) The MS/MS spectrum of NPM1 is shown.

**
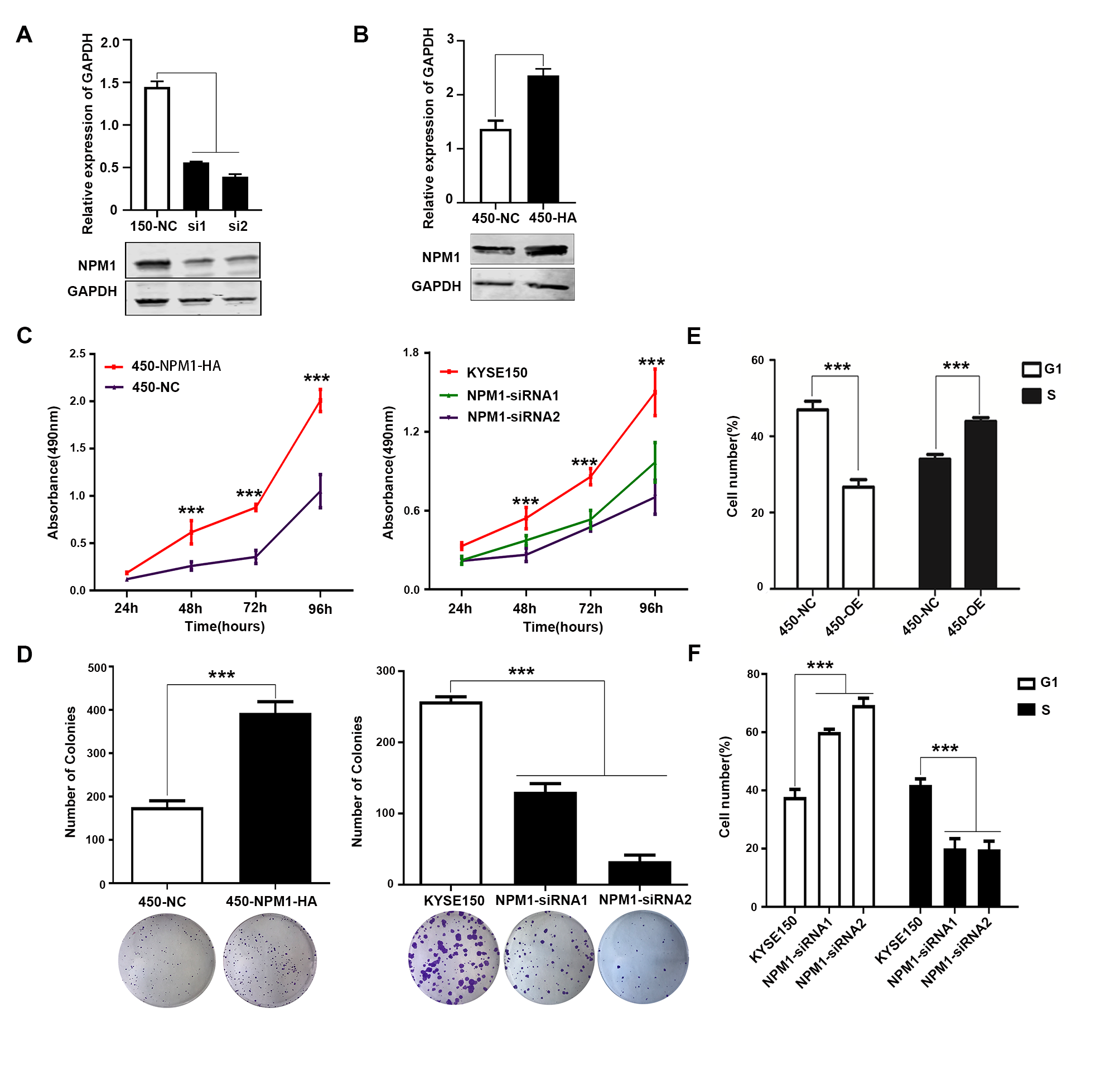
 Fig S5 The expression of NPM1 effects ESCC cell proliferation, cell cycle,**

(A-B), Detected the efficiency of knock-down (A) and over-expression (B) NPM1 protein in KYSE150 and KYSE450 cells by western blot. (C) The expression of NPM1 effected the ability of proliferation in KYSE150 and KYSE450 cells. (D) The expression of NPM1 effected the ability of colony formation in KYSE150 and KYSE450 cells. (E-F) The expression of NPM1 effected the cell cycle by flow cytometry assay in KYSE150 and KYSE450 cells. All data are presented as the mean±standard deviation and are repeated in three independent experiments. Statistical analysis is performed with one-way ANOVA. ****P* < 0.001.

**
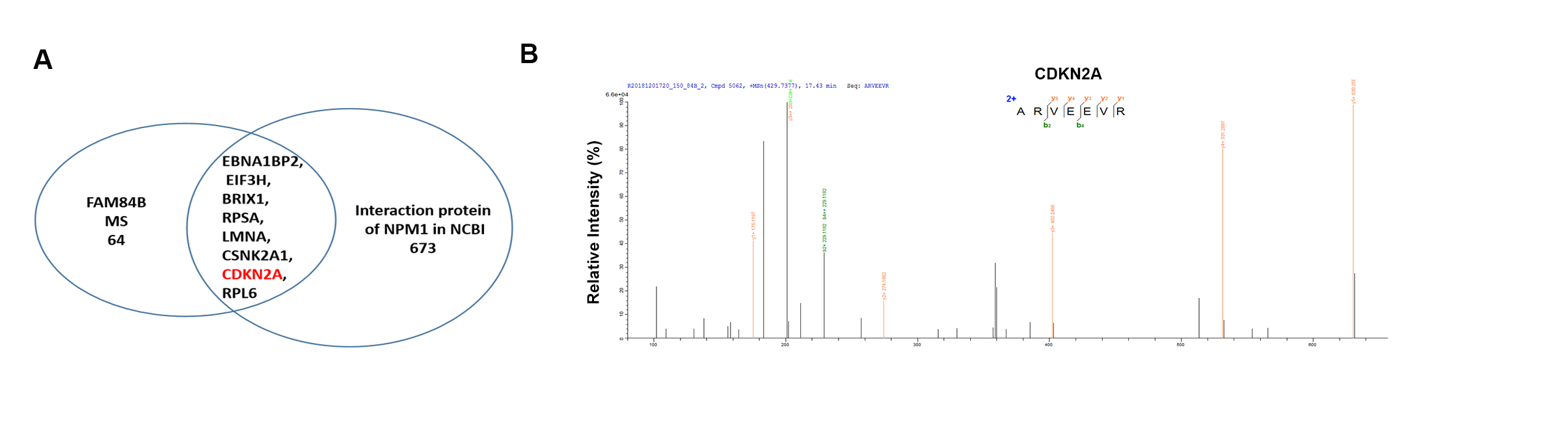
**

**Fig S6 The result of CDKN2A via MS/MS spectrum**

(A) The CDKN2A as potential targets of combinding with FAM84B and NPM1 simultaneously was filtered the MS results of FAM84B and the proteins interacting with NPM1 in NCBI database. (B) The MS/MS spectrum of CDKN2A is shown.


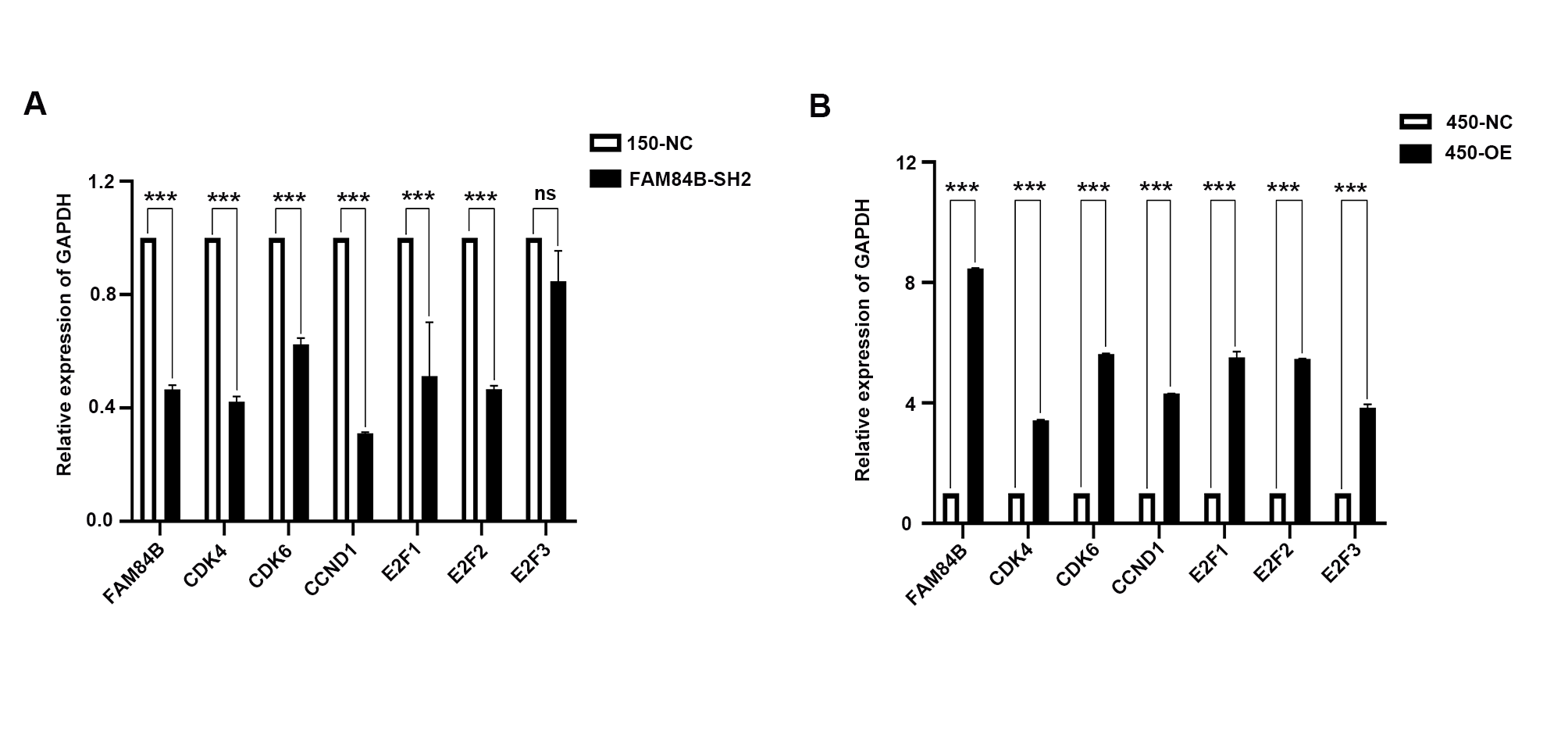


**Fig S7 Deteced the mRNA expression of cell cycle proteins in over-expression/knock down FAM84B cells.**

(A) Knock-down FAM84B decreased the mRNA expression of cell cycle proteins. (B) Over-expression FAM84B increased the mRNA expression of cell cycle proteins.


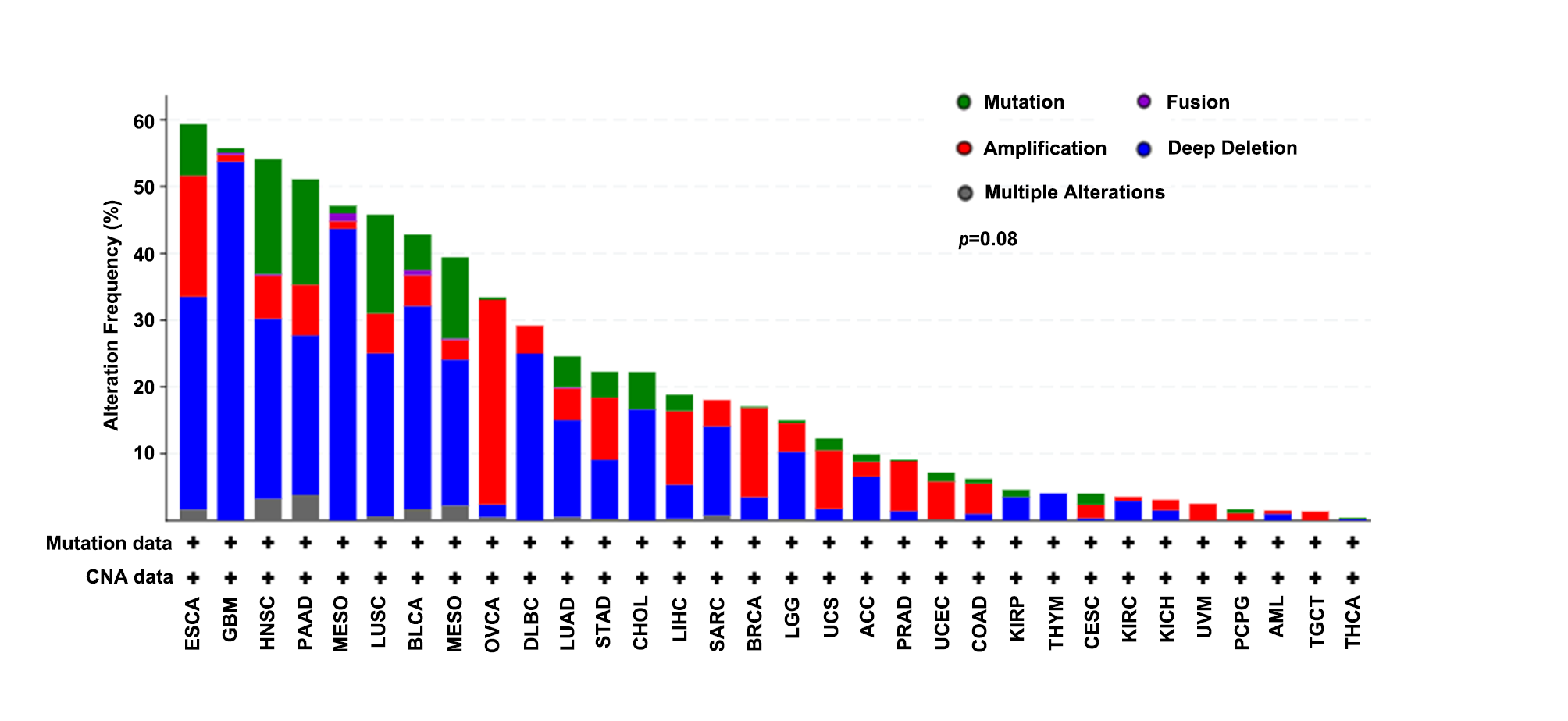


**Fig S8 The mutations of FAM84B and CDKN2A in various cancer types**
